# Inferior Turbinate Abscess from Bulb Suctioning in a Pediatric Patient

**DOI:** 10.1002/oto2.140

**Published:** 2024-04-26

**Authors:** Caden D. Duffy, Madeleine E. Gallagher, Paul W. Bauer, Maxie D. Brewer

**Affiliations:** ^1^ Anne Burnett Marion School of Medicine Texas Christian University Fort Worth Texas USA; ^2^ Cook Children's Medical Center Fort Worth Texas USA

**Keywords:** bulb suction, inferior turbinate, nasal abscess, pediatric facial edema

The inferior turbinates are the largest of the 3 pairs of turbinates, playing a role in the humidification, directing, and filtering of air. Enlarged inferior turbinates can occur from environmental exposures such as cigarette smoking, allergies, and chronic rhinosinusitis. Enlargement due to abscess formation is highly unusual. Other abscesses of the nasal cavity can infrequently occur, usually caused by nasal trauma, surgery, or dental infections. Irritation and trauma results in blood vessel rupture and hematoma formation, allowing a static environment ideal for bacteria and abscess creation. Abscesses have been well reported in septoplasty literature with rates of infection and abscess formation widely ranging from 0.4% to 12% postoperatively.^1^ To the authors' knowledge, there have been no previous reports of an inferior turbinate abscess in an otherwise healthy pediatric patient.

## Case Presentation

A previously healthy 4‐month‐old male presented to our emergency department with right‐sided facial swelling. This began 6 days previously, accompanied by an unremitting fever and bilateral nasal congestion with rhinorrhea. His parents denied recent nasal trauma but did report frequent use of bulb suctioning secondary to worsening congestion. Patient was up to date on his vaccinations and had no previous medical conditions, hospitalizations, or surgeries.

On physical exam, the infant had right‐sided rhinorrhea and nasal swelling at the right nasal base. The nasal cavity was filled with thick purulent secretions and erythematous soft tissue. The left nasal cavity was unremarkable. His intraoral examination revealed black discoloration of his anterior gingiva in the area directly inferior to the nasal vestibule.

Laboratory work showed a leukocytosis of 20,670 cubic millimeters, with slight elevation of procalcitonin and C‐reactive protein. IgG, IgM, IgA, and IgE levels were normal and a lymphocyte subset panel had appropriate elevations. A CT of his facial bones showed a rim‐enhanced collection protruding from the anterior right nasal cavity, measuring approximately 1.7 × 0.9 × 1.2 cm, with regional soft tissue thickening and enhancement consistent with a soft tissue abscess.

**Figure 1:**
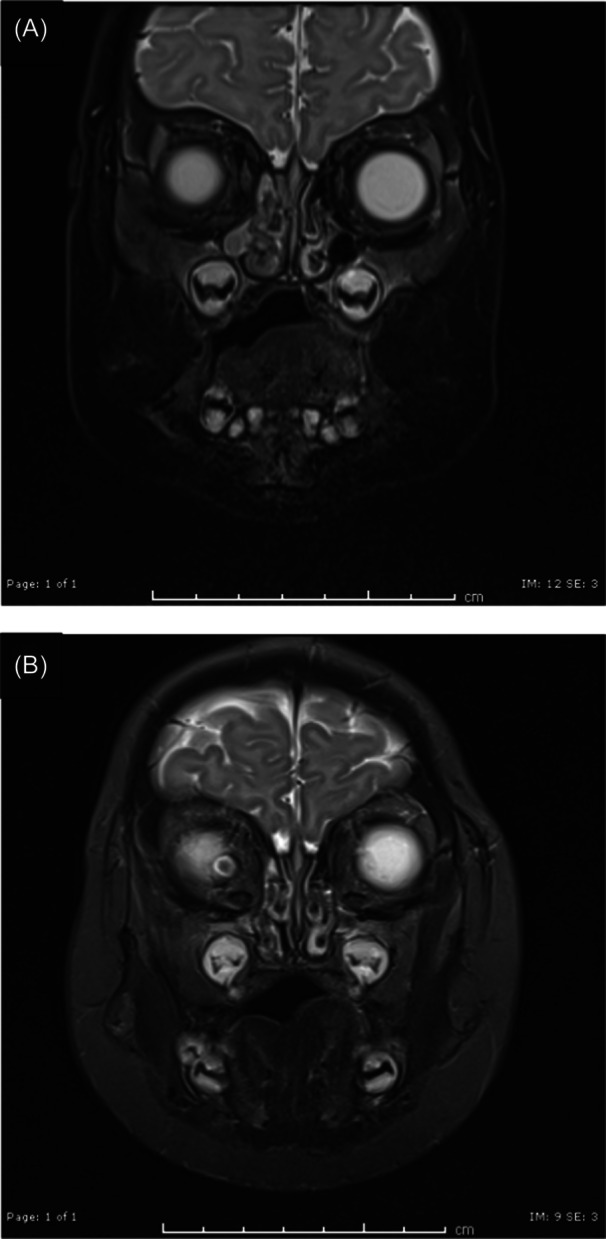
(A) Magnetic resonance showing T2‐weighted imaging obtained upon admission to the hospital. Fluid collection seen with features consistent with an abscess adjacent to the right inferior turbinate. (B) Magnetic resonance showing T2‐weighted imaging obtained 4 days after incision and drainage, confirming the resolution of the abscess and no indication of further complications.

**Figure 2:**
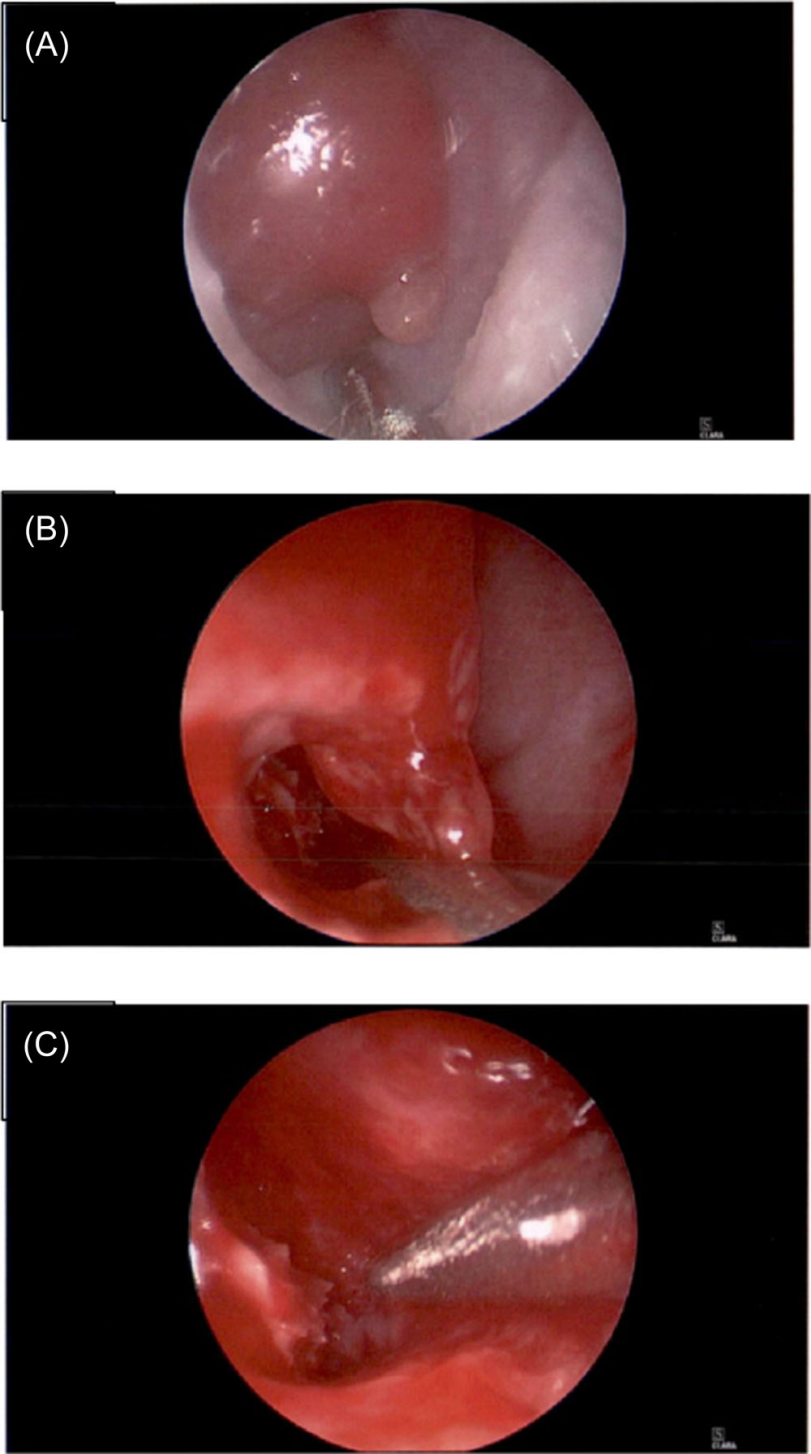
(A) Endoscopic view at start of procedure showing the anterior margin of the right nasal cavity including the inferior turbinate. (B) Endoscopic view looking into the right inferior turbinate once the abscess has been incised and drained. Inferior conchae bone is visualized. (C) Endoscopic view inside of the right inferior turbinate. The length of the inferior conchae bone is well visualized.

Upon admission, a head magnetic resonance imaging (MRI) was performed showing multiple fluid collections in the right nasal cavity with associated restriction of diffusion, consistent with the presence of purulent material forming an abscess (Figure 1A). There was no evidence of a congenital anomaly or intracranial extension. The patient underwent nasal endoscopy, which noted friable granulation tissue in the right lateral and anterior vestibule, with an abscessed right inferior turbinate (Figure 2A). Pus was released, aspirated, and sent for cultures, and the nasal cavity was irrigated with saline (Figure 2B). Once the abscess was drained, the endoscope could be advanced within the inferior turbinate revealing exposed inferior turbinate bone with no attached soft tissue (Figure 2C). The granulated mass was biopsied with pathology showing evidence of respiratory mucosa along with fibrinopurulent and necrosed material. Cultures of the abscess grew methicillin‐resistant *Staph aureus* sensitive to clindamycin.

A repeat MRI was ordered 4 days after surgery due to concerns for exposed bone of the inferior turbinate, the black discoloration of the contiguous gingiva and mild residual swelling. This imaging showed resolution of the abscess and no evidence of osteomyelitis (Figure 1B). The patient was discharged home the next day with instructions to complete a 14‐day course of clindamycin and to switch from bulb suctioning to nasal aspiration to minimize additional nasal trauma.

## Discussion

Given a history of frequent bulb suctioning, we highly suspect the etiology of this nasal abscess was iatrogenic. Although occasional bulb suctioning is effective at clearing the nasal cavities, being overly aggressive can be harmful. Several factors have been reported to impact how nasal suction is used, such as whether hand placement is radial or axial in position and differences in use between the sexes.^2^ Alternatives such as a nasal aspirator may also be a safer option, although further comparison studies are needed.

Although a CT was initially performed to define the abscess and look for bony erosion, an MRI was required to assess for an anterior cephalocele, nasal dermal sinus cyst, tract, or fistula, or nasal neural glial heterotopia and were ruled out. Patient was subsequently taken to the OR for incision and drainage with cultures obtained to define the infecting organism and to direct treatment.

## Author Contributions

**Caden D. Duffy**, wrote and revised the article; **Madeleine E. Gallagher**, wrote and revised the article; **Paul W. Bauer**, designed, wrote, and revised the article; **Maxie D. Brewer**, designed, wrote, and revised the article.

## Disclosures

### Competing interests

None.

### Funding source

None.
